# Determinants of self-reported functional status (EPIC-26) in prostate cancer patients prior to treatment

**DOI:** 10.1007/s00345-020-03097-z

**Published:** 2020-02-10

**Authors:** Rebecca Roth, Sebastian Dieng, Alisa Oesterle, Günter Feick, Günther Carl, Andreas Hinkel, Thomas Steiner, Björn Theodor Kaftan, Frank Kunath, Boris Hadaschik, Simba-Joshua Oostdam, Rein Jüri Palisaar, Mateusz Koralewski, Burkhard Beyer, Björn Haben, Igor Tsaur, Simone Wesselmann, Christoph Kowalski

**Affiliations:** 1 Institute of Medical Statistics and Computational Biology (IMSB), Medical Faculty, University of Cologne, Kerpener Straße 62, 50937 Cologne, Germany; 2 OnkoZert, Gartenstraße 24, 89231 Neu-Ulm, Germany; 3 Federal Association of German Prostate Cancer Patient Support Groups, Thomas-Mann-Str. 40, 5311 Bonn, Germany; 4 Help for Prostate Cancer Patients (Förderverein Hilfe Bei Prostatakrebs e.V., FHbP), Louise Schroeder Ring 2, 25436 Tornesch, Germany; 5 Franziskus Hospital, Kiskerstraße 26, 33615 Bielefeld, Germany; 6 Helios Klinikum Erfurt, Nordhäuser Straße 74, 99089 Erfurt, Germany; 7 Städtisches Klinikum Lüneburg, Bögelstraße 1, 21339 Lüneburg, Germany; 8 Department of Urology and Pediatric Urology, University Hospital Erlangen, FAU Erlangen-Nürnberg, Krankenhausstraße 12, 91052 Erlangen, Germany; 9 Klinik und Poliklinik für Urologie, Kinderurologie und Uroonkologie, Universitätsklinikum Essen (AöR), Hufelandstraße 55, 45147 Essen, Germany; 10 Vinzenz-Krankenhaus Hannover, Lange-Feld-Str. 31, 30559 Hannover, Germany; 11 Urologische Klinik, Marien Hospital Herne, Universitätsklinikum der Ruhr-Universität Bochum, Mitten in der ST. ELISABETH GRUPPE GmbH, Katholische Kliniken Rhein-Ruhr, Widumer Str. 8, Herne, 44627 Germany; 12 Urologie, Krankenhaus der Barmherzigen Brüder Trier, Nordallee 1, 54292 Trier, Germany; 13 Martini-Klinik Prostate Cancer Center Hamburg, Martinistraße 52, 20246 Hamburg, Germany; 14 St. Marien Hospital Ahaus, Wüllener Str. 101, 48683 Ahaus, Germany; 15 Klinik und Poliklinik für Urologie und Kinderurologie, Universitätsmedizin der Johannes Gutenberg-Universität Mainz, Langenbeckstraße 1, 55131 Mainz, Germany; 16 German Cancer Society, Kuno-Fischer-Straße 8, 14057 Berlin, Germany

**Keywords:** Health-service research, Prostate neoplasms, Patient-reported outcome measures, Functional status, Case-mix adjustment, Multilevel analysis

## Abstract

**Purpose:**

The self-reported functional status (sr-FS) of prostate cancer (PCa) patients varies substantially between patients and health-care providers before treatment. Information about this issue is important for evaluating comparisons between health-care providers and to assist in treatment decision-making. There have been few reports on correlates of pretherapeutic sr-FS. The objective of the article, therefore, is to describe clinical and sociodemographic correlates of pretherapeutic sr-FS, based on a subset of the TrueNTH Global Registry, a prospective cohort study.

**Methods:**

A total of 3094 PCa patients receiving local treatment in 44 PCa centers in Germany were recruited between July 2016 and April 2018. Multilevel regression models were applied to predict five pretherapeutic sr-FS (EPIC-26) scores based on clinical characteristics (standard set suggested by the International Consortium for Health Outcomes Measurement), sociodemographic characteristics, and center characteristics.

**Results:**

Impaired pretherapeutic sr-FS tended to be associated with lower educational level and poorer disease characteristics—except for “urinary incontinence” which was only associated with age. Notably, age was a risk factor (“urinary incontinence,” “urinary irritative/obstructive,” “sexual”) as well as a protective factor (“hormonal”) for pretherapeutic sr-FS. Pretherapeutic sr-FS varies little across centers.

**Conclusions:**

Pretherapeutic sr-FS varies by clinical patient characteristics and age as well as by socioeconomic status. The findings point out the benefit of collecting and considering socioeconomic information in addition to clinical and demographic patient characteristics for treatment decision-making and fair comparisons between health-care providers.

**Electronic supplementary material:**

The online version of this article (10.1007/s00345-020-03097-z) contains supplementary material, which is available to authorized users.

## Introduction

Improving the quality of health care requires information about patient outcomes. In addition to clinical outcomes such as survival, the importance of patient-reported outcome measures (PROMs) has increasingly been acknowledged in recent years [1]. PROMs are not only assessed in clinical trials [2], but also in routine care, for two major purposes: first, to assist in clinical decision-making and to monitor patients’ health over time [3, 4]; second, for reasons of quality assurance and more specifically, to compare performance between health-care providers [5]. PROMs are particularly important in care for patients with prostate cancer (PCa) since the chances of long-term survival are very good [6], whereas impairment of functional status (FS) may be substantial [7]. Many initiatives such as the Michigan Urological Surgery Improvement Collaborative and the Martini-Klinik program have already implemented collection of PROM data in routine care for PCa patients [8, 9]. However, such approaches are often isolated or involve programs that are limited to single locations or regions.

In PCa patients, FS not only results from the treatment received but also varies substantially, long before treatment [10]. With the emergence of PROM assessment, information about this issue has become important for evaluating fair comparisons between health-care providers, identifying groups of patients who are at risk for impaired FS, and making treatment decisions accordingly. However, there have been few reports to date on correlates of pretherapeutic FS [11]. In particular, there is a lack of studies that go beyond clinical characteristics and include measures of socioeconomic status, although social determinants of health are high on the global public health agenda [12].

This paper is based on a multicenter sample of PCa patients receiving treatment from prostate cancer centers (PCCs) in Germany. The data were collected as part of the ongoing Prostate Cancer Outcomes (PCO) study [13]. The PCO study is part of the Movember-funded TrueNTH registry, which is collecting and harmonizing patient data in more than 15 countries worldwide [14]. The primary objective of the exploratory investigation presented in this article is the description of correlates, in terms of patient and center characteristics, with pretherapeutic self-reported FS (sr-FS).

## Patients and methods

### Data collection

Following approval of the study by the local ethics committee of the Medical Association of Berlin (Eth-12/16), PCCs certified by the German Cancer Society [15] were invited to participate in the PCO Study (DRKS00010774), an ongoing prospective, population-based cohort study [13]. Starting in July 2016, all PCa patients with a first diagnosis (any T, any N, M0) at one of the participating centers in Germany who were receiving local treatment as well as patients scheduled for active surveillance (AS) and watchful waiting (WW) were eligible for inclusion. Patients were excluded if they were unable to administer a questionnaire. After giving informed consent, participants were asked to answer a baseline (i.e., pretherapeutic) questionnaire within 3 months after diagnosis for patients scheduled for AS or WW afterwards or at least 3 months before local treatment. During the study, an annual follow-up questionnaire will be administered at least once. The analysis presented here is based on the pretherapeutic assessment. The pretherapeutic questionnaire includes items in accordance with the International Consortium for Health Outcomes Measurement (ICHOM) standard set for localized PCa [16], with three additional items (see “Measures”). The data were matched with clinical and treatment data.

### Measures

The questionnaire includes the five Expanded Prostate Cancer Index Composite 26-item version (EPIC-26) domain scores on sr-FS calculated in accordance with the scoring instructions [17, 18]. The domains for “urinary incontinence” and “urinary irritative/obstructive” consist of four items, for “hormonal” of five items, and for “bowel” and “sexual” of six items [17]. Lower scores (on a range of 0–100) indicate poorer function. Sociodemographic information was collected using items from the standard questionnaire used by the German pension insurance fund: highest school-leaving certificate (lower secondary school, intermediate secondary school, entrance certificate for a higher technical college/university of applied science, university entrance certificate, other, none); nationality (German with/or without another nationality, not German); health-insurance status (statutory, private). Patients with health insurance other than statutory or private (*n* = 11, 0.4%) were excluded due to small patient numbers. Age (continuous) and disease and treatment information were documented by the hospital.

Risk classification at baseline (localized PCa with low/intermediate/high risk, locally advanced PCa, advanced PCa; Online Resource 10) was coded according to the German clinical guideline [19, p. 60] combining prostate-specific antigen level, Gleason score, and clinical stage. Numbers of comorbidities at baseline were coded as 0, 1, or ≥ 2 [16]. The type of first treatment initiated after questionnaire administration was grouped as radiotherapy (RT), radical prostatectomy (RPE), AS, and WW. Patients with other local treatments after questionnaire administration, e.g., radical cystoprostatectomy, and high-intensity focused ultrasound, were excluded (*n* = 14, 0.5%) as well as patients who had received androgen deprivation therapy, AS, or WW before questionnaire administration (*n* = 196, 6.3%).

PCC characteristics were urban status (small- to medium-sized town/large city/metropolitan city, i.e. ≤ 100,000/ > 100,000–1,000,000/ > 1,000,000 inhabitants), the center’s teaching status (university hospital/teaching hospital/nonteaching hospital), hospital ownership status (public/private/charitable), and recruitment rate (i.e., the rate of participating patients divided by the number of eligible patients at the respective PCC).

### Statistical analysis

Descriptive analyses present means, standard deviation (SD), medians, interquartile ranges, and ranges for continuous variables, or numbers and percentages for categorical variables. In exploratory analyses, for each domain separately, associations with pretherapeutic sr-FS (outcome) were investigated using multivariable linear multilevel models to account for confounding and for the clustering of patients (level-1) in PCCs (level-2). A null model was used to calculate the intraclass correlation coefficient (ICC), i.e., the proportion of variance in pretherapeutic sr-FS explained by the variation between PCCs. Model 1 contains sociodemographic predictors only (independent variables), model 2 additionally comprises baseline disease information (independent variables), and model 3 also contains PCC characteristics (independent variables). Centers that recruited fewer than 10 patients were pooled, i.e., data were analyzed as if belonging to one common center. Values for PCC-level variables for pooled centers were calculated as weighted averages of center-specific variable values, where the center-specific relative sample size was used as the weighting. Missing data were not imputed. For categorical variables, missing categories were included in the multilevel models. The following sensitivity analyses were performed to investigate the robustness of the results of the multilevel analyses for model 3 with respect to a deviation from the assumption of normality of residuals: first, bootstrapping (10,000 samples); second, multilevel analyses for rankit-transformed scores (aiming to achieve normality); third, generalized multilevel analyses using penalized quasi-likelihood, assuming a gamma distribution of scores (not assuming normality). Further sensitivity analyses for model 3 were performed by excluding centers that did not document comorbidities, and with additional adjustment for the type of treatment to account for differences in recruitment according to the different specialists (urologists/radiotherapists) and treatment provided.

As this is an exploratory study, no correction for multiple testing was performed. *P* values < 0.05 were regarded as statistically significant. Statistical computations were carried out using the R program [20]. The lme4 package was used for multilevel analysis [21]. Generalized linear multilevel models were run using the MASS package [22].

## Results

Data of 44 centers and 3094 patients who were recruited between July 1, 2016, and March 29, 2018, were collected. Table 1 presents descriptive analyses of the patients and centers that were included in multilevel analyses after exclusion of patients as reported above and those with missing information (*n* = 220 (7%) patients excluded; Online Resource 1). The lowest pretherapeutic sr-FS was observed for the “sexual” domain (mean ± SD = 58.95 ± 29.30), the highest pretherapeutic sr-FS was noted for the “bowel” domain (mean ± SD = 95.96 ± 9.56). The percentage of missing pretherapeutic sr-FS for individual domains ranged between 4.4% (“sexual”) and 8.7% (“urinary irritative/obstructive”). The percentage of missing values was low for predictor variables (0–5%), except for the number of comorbidities (21.6%), which is mainly attributable to 14 centers in which comorbidities were not documented. For multilevel analyses, two centers (10 patients) were pooled. One center (21 patients) was excluded from analyses of model 3 due to a missing recruitment rate.

**Table 1:** Descriptive statistics for patient characteristics—pretherapeutic functional status and predictor variables

Variable	Mean ± SD	Median (IQR)	Range
**Pretherapeutic functional status**			
Urinary incontinence (missing: 6.3%; *n* = 193)	92.39 ± 14.54	100 (91.75–100)	0–100
Urinary irritative/obstructive (missing: 8.7%; *n* = 269)	85.55 ± 15.95	87.5 (75–100)	0–100
Bowel (missing: 8.3%; *n* = 257)	95.96 ± 9.56	100 (95.83–100)	29.17–100
Sexual (missing: 4.4%; *n* = 135)	58.95 ± 29.3	62.5 (33.4–87.5)	0–100
Hormonal (missing: 6.2%; (*n* = 192)	90.05 ± 14.49	95 (85–100)	0–100
**Patient characteristics**			
*Sociodemographic information*			
Age (missing: 0%; *n* = 0)	66 ± 7	66 (61–72)	39–85

	%	*n*	
Nationality (missing: 4.7%; *n* = 145)			
German	91.6	2632	
Other	3.6	104	
Health insurance (missing: 4.7%; *n* = 146)			
Statutory	73.6	2115	
Private	21.6	620	
School-leaving qualification (missing: 5%; *n* = 154)			
Lower secondary school	37.2	1069	
Intermediate secondary school	23.5	675	
FHSR	11.8	339	
University entrance certificate	20.1	578	
Other	1.6	45	
None	0.7	20	
*Disease information*			
Comorbidities (missing: 21.6%; *n* = 666)			
0	49	1408	
1	22.5	647	
≥ 2	7.3	210	
Risk class ^a^ (missing: 0%; *n* = 0)			
High, localized	32.2	924	
Intermediate, localized	46.1	1325	
Low, localized	15.9	457	
Locally advanced	4.7	136	
Advanced	1.1	32	
*Treatment allocation*			
Treatment initiated after questionnaire administration(missing: 0%; *n* = 0)			
RPE	93.1	2676	
RT	5.5	159	
AS	1.1	31	
WW	0.3	8	

Center characteristics	Mean ± SD	Median (IQR)	Range
Recruitment rate (missing: 0.7%; *n* = 21)	50.92 ± 17.08	56 (38–62)	5–95
	%	n	
Ownership (missing: 0%; *n* = 0)			
Charitable	3.0	86	
For profit	51.5	1481	
Public	45.5	1307	
Urban status (missing: 0%; *n* = 0)			
≤ 100,000	34.5	992	
> 100,000–1,000,000	59.5	1710	
> 1,000,000	6.0	172	
Teaching status (missing: 0%; *n* = 0)			
No	4.1	117	
Yes, non-university	79.8	2293	
Yes, university	16.1	464	

^a^Risk class in accordance with the German clinical guideline [28]

*AS* active surveillance, *FHSR* *Fachhochschulreife,* entrance certificate for a higher technical college/university of applied science, *IQR* interquartile range, *RPE* radical prostatectomy, *RT* radiotherapy, *SD* standard deviation, *WW* watchful waiting

The results of the multilevel analyses were consistent across models 1 (Online Resource 2), 2 (Online Resource 3), and 3 (Table 2). No uniform patterns emerged across the five EPIC-26 domains (Table 2); with regard to sociodemographic factors, older age was associated with poorer pretherapeutic functioning for “urinary incontinence”, “urinary irritative/obstructive”, and—most strongly—“sexual”—i.e., with each year of age, the pretherapeutic “sexual” sr-FS is reduced by 1.38 points. By contrast, older age was related to an improved pretherapeutic “hormonal” sr-FS. Higher educational levels were associated with better pretherapeutic sr-FS in the “hormonal” domain and more strongly in the “sexual” domain, e.g., sr-FS was 8.34 higher for patients with a university entrance certificate compared to patients with lower secondary school-leaving certificates. For the “irritative/obstructive” and “bowel” domains, no school-leaving certificate versus a lower secondary school certificate was associated with significantly lower pretherapeutic sr-FS.

**Table 2:** Results of linear multilevel analyses for self-reported pretherapeutic functional status (model 3)

	Urinary incontinence (2679 patients, 42 centers)	Urinary irritative/obstructive (2612 patients, 42 centers)	Bowel (2617 patients, 42 centers)	Sexual (2729 patients, 42 centers)	Hormonal (2678 patients, 42 centers)
	Estimate (95% CI); *p* value	Estimate (95% CI); *p* value	Estimate (95% CI); *p* value	Estimate (95% CI); *p* value	Estimate (95% CI); *p* value
(Intercept)	104.35 (98.68 to 110.02)	95.67 (89.34 to 101.99)	95.5 (91.83 to 99.16)	149.06 (138.71 to 159.4)	75.45 (69.99 to 80.92)
**Patient characteristics**					
*Sociodemographic information*					
Age	**− 0.19 (− 0.27 to − 0.11);** < 0.001*	**− 0.13 (− 0.21 to − 0.04);** 0.004*	0 (− 0.05 to 0.05); 0.905	**− 1.38 (− 1.53 to − 1.24);** < 0.001*	**0.25 (0.17 to 0.32);** < 0.001*
Nationality (reference: German)					
Other	− 2.18 (− 5.30 to 0.94); 0.170	− 2.94 (− 6.37 to 0.49); 0.093	0.13 (− 1.91 to 2.18); 0.898	0.47 (− 5.30 to 6.24); 0.872	0.47 (− 2.56 to 3.5); 0.760
Insurance (reference: statutory)					
Private	1.18 (− 0.26 to 2.62); 0.109	1.27 (− 0.33 to 2.86); 0.119	0.72 (− 0.24 to 1.67); 0.140	2.44 (− 0.21 to 5.08); 0.071	1.28 (− 0.13 to 2.70); 0.076
School-leaving qualification (reference: lower secondary school)					
Intermediate secondary school	1.35 (− 0.13 to 2.84); 0.074	0.69 (− 0.96 to 2.34); 0.411	0.34 (− 0.65 to 1.33); 0.505	**3.45 (0.75 to 6.16);** 0.012*	**2.16 (0.69 to 3.63);** 0.004*
FHSR	0.94 (− 0.92 to 2.79); 0.323	− 0.26 (− 2.31 to 1.79); 0.803	− 0.77 (− 2.01 to 0.48); 0.226	**6.40 (2.99 to 9.81);** < 0.001*	− 0.09 (− 1.94 to 1.76); 0.921
University entrance certificate	0.74 (− 0.89 to 2.37); 0.375	0.18 (− 1.63 to 1.98); 0.849	0.18 (− 0.91 to 1.27); 0.746	**8.34 (5.34 to 11.34);** < 0.001*	**2.01 (0.41 to 3.62);** 0.014*
Other	0.64 (− 3.93 to 5.21); 0.784	− 2.43 (− 7.48 to 2.61); 0.344	0.95 (− 2.00 to 3.90); 0.529	− 1.45 (− 9.80 to 6.90); 0.733	1.53 (− 2.95 to 6.00); 0.504
None	− 0.58 (− 7.42 to 6.26); 0.869	**− 10.46 (− 17.93 to − 3.00);** 0.006*	**− 4.70 (− 9.01 to − 0.40);** 0.032*	− 7.85 (− 19.92 to 4.22); 0.202	0.76 (− 5.86 to 7.39); 0.821
*Disease information*					
Comorbidities (reference: 0)					
1	0.34 (− 1.12 to 1.79); 0.652	0.67 (− 0.96 to 2.31); 0.419	− 0.40 (− 1.34 to 0.55); 0.412	**− 4.82 (− 7.50 to − 2.15);** < 0.001*	**− 2.16 (− 3.56 to − 0.75);** 0.003*
≥ 2	1.17 (− 1.08 to 3.42); 0.308	− 1.63 (− 4.14 to 0.87); 0.201	**− 1.71 (− 3.19 to − 0.23);** 0.023*	− 8.80 (− 12.96 to − 4.64); < 0.001*	**− 4.38 (− 6.58 to − 2.17)** < 0.001*
Risk class^a^ (reference: low, localized)					
High, localized	− 0.44 (− 2.15 to 1.27); 0.616	**− 2.12 (− 4.01 to − 0.22);** 0.029*	**− 1.33 (− 2.48 to − 0.19);** 0.023*	− 0.89 (− 4.03 to 2.25); 0.578	**− 1.97 (− 3.66 to − 0.27);** 0.023*
Intermediate, localized	0.56 (− 1.06 to 2.18); 0.495	− 0.13 (− 1.93 to 1.67); 0.889	− 0.76 (− 1.85 to 0.32); 0.169	1.85 (− 1.12 to 4.83); 0.222	− 0.37 (− 1.98 to 1.24); 0.654
Locally advanced	1.32 (− 1.61 to 4.25); 0.377	**− 3.48 (− 6.71 to − 0.24);** 0.035*	− 1.12 (− 3.05 to 0.82); 0.259	− 1.01 (− 6.31 to 4.29); 0.710	− 1.35 (− 4.26 to 1.57); 0.365
Advanced (N1)	− 1.99 (− 7.37 to 3.38); 0.468	**− 13.39 (− 19.36 to − 7.41);** ** < 0.001**	**− 5.32 (− 8.93 to − 1.71);** 0.004*	− 8.05 (− 18.01 to 1.91); 0.113	− 0.98 (− 6.15 to 4.18); 0.709
**Center characteristics**					
Recruitment rate	− 1.12 (− 5.15 to 2.91); 0.586	0.06 (− 4.69 to 4.80); 0.982	**2.60 (0.20 to 5.01);** 0.034*	− 0.37 (− 7.52 to 6.77); 0.918	− 2.11 (− 5.64 to 1.42); 0.241
Ownership (reference: public)					
Charitable	0.25 (− 3.98 to 4.48); 0.908	− 1.27 (− 6.17 to 3.63); 0.612	**− 2.68 (− 5.19 to − 0.17);** 0.037*	− 2.01 (− 9.43 to 5.41); 0.595	− 2.73 (− 6.44 to 0.98); 0.149
For profit	− 0.17 (− 2.10 to 1.77); 0.865	0.12 (− 2.17 to 2.40); 0.920	− 1.01 (− 2.15 to 0.13); 0.083	− 0.65 (− 4.05 to 2.75); 0.708	− 0.59 (− 2.27 to 1.09); 0.489
Urban status (reference: ≤ 100,000)					
> 100,000–1,000,000	0.24 (− 1.67 to 2.15); 0.805	− 1.42 (− 3.69 to 0.84); 0.218	**1.14 (0.02 to 2.25);** 0.046*	− 0.34 (− 3.69 to 3.00); 0.841	1.50 (− 0.15 to 3.14); 0.075
> 1,000,000	− 1.68 (− 5.73 to 2.37); 0.415	− 3.63 (− 8.31 to 1.06); 0.129	0.79 (− 1.64 to 3.23); 0.524	− 0.01 (− 7.34 to 7.32); 0.998	2.53 (− 1.17 to 6.23); 0.181
Teaching (reference: yes, non-university)					
No	0.40 (− 2.92 to 3.71); 0.815	**− 4.05 (− 7.92 to − 0.17);** 0.041*	1.30 (− 0.73 to 3.32); 0.209	− 2.99 (− 8.85 to 2.88); 0.318	− 0.93 (− 3.89 to 2.02); 0.536
Yes, university	− 1.18 (− 3.78 to 1.41); 0.371	0.39 (− 2.69 to 3.47); 0.805	**− 1.55 (− 3.02 to − 0.08);** 0.038*	− 1.18 (− 5.72 to 3.36); 0.610	− 0.93 (− 3.10 to 1.25); 0.404

FHSR, *Fachhochschulreife,* entrance certificate for a higher technical college/university of applied science

*Bold estimates, confidence intervals, and *p* values indicate *p* values < 0.05

1. Multilevel model 3 accounts for the clustering of patients (level-1) in prostate cancer centers (level-2) with a random intercept. Fixed effects for sociodemographic predictors, disease information, and prostate cancer center characteristics are included

2. Patient numbers vary between the analyses of different domains due to different numbers of missing values for the respective scores

3. Results for the missing categories for categorical variables are not presented in this table

^a^Risk class in accordance with the German clinical guideline [19]

With regard to clinical predictors, comorbidities were associated with lower pretherapeutic sr-FS in the “bowel”, “sexual”, and “hormonal” domains. Most notably, in the “sexual” domain, patients with two or more comorbidities scored 8.80 points lower than patients without comorbidities. In addition, some higher risk classes, i.e., risk class “localized PCa with high risk” and higher, were associated with poorer pretherapeutic sr-FS in the “bowel”, “hormonal” and most pronounced in the “urinary irritative/obstructive” domain; the sr-FS was reduced by 13.39 points for patients with advanced PCa versus localized PCa with low risk.

Variation in pretherapeutic sr-FS across centers was low [range of ICCs in the null models: < 0.001 (“bowel”) to 0.024 (“sexual”); Online Resource 4] and decreased further after controlling for patient-level variables (i.e., for model 1 and 2).

With regard to center characteristics (Table 2), in the “bowel” domain, higher recruitment was associated with higher pretherapeutic sr-FS and was also observed for large cities versus small- to medium-sized towns. By contrast, pretherapeutic sr-FS in the “bowel” domain was lower in PCCs of charitable versus public ownership and in university versus non-university teaching hospitals. A decreased pretherapeutic sr-FS was also observed in the “urinary irritative/obstructive” domain for nonteaching versus non-university teaching hospitals.

The results of the sensitivity analyses largely confirmed the results of the main analyses of model 3 (Online Resources 5–9). In the sensitivity analyses after adjustment for the type of treatment, associations were detected between treatment decision and pretherapeutic function; in comparison with treatment with RPE, RT was associated with poorer sr-FS in the “hormonal” domain, i.e., sr-FS was 3.18 points lower for patients treated with RT compared to RPE. AS was associated with poorer sr-FS in the “urinary incontinence” and “urinary irritative/obstructive” domains. WW was associated with poorer sr-FS in the “urinary incontinence” and “bowel” domains.

## Discussion

Correlates of pretherapeutic sr-FS are rarely discussed in the literature. However, knowledge about such associations is of importance for clinicians to more easily identify patients at risk for impaired function and to consider this in treatment decision-making. This is especially true since sr-FS is not only associated with clinical characteristics and age—a matter of given for every provider—but also with socioeconomic status, as shown in this study. In addition, findings may sharpen practitioners understanding of why patient-specific information is needed to allow for fair comparisons between health-care providers. This paper provides such information for pretherapeutic sr-FS from a large multicenter population of PCa patients in Germany. Correlates of pretherapeutic sr-FS were sociodemographic and clinical patient characteristics: age was a risk factor for impaired sr-FS in the “urinary incontinence,” “urinary irritative/obstructive,” and “sexual” domains and a protective factor in the “hormonal” domain—a finding that corresponds to the results of a prospective cohort study in the United States [23]. The latter association may, however, be a result of perception rather than a true protective effect of age, i.e., aging men during andropause may already have suffered from corresponding symptoms some time before PCa diagnosis and may, therefore, have adjusted to symptoms [24]. In clinical practice, when counseling patients, interpretation of the presented results may also benefit from the results of Laviana et al. who broke down the domain scores into functional outcomes to better interpret the domain scores [25]. In the present analyses, impaired pretherapeutic sr-FS correlated with lower educational level (“urinary irritative/obstructive,” “bowel,” “sexual,” “hormonal”), comorbidities (“bowel,” “sexual,” “hormonal”; comparable results reported in [23]), and higher risk classes (i.e., risk classes “localized PCa with high risk” and higher; “urinary irritative/obstructive,” “bowel”, “hormonal”). Sensitivity analyses showed, i.a., that pretherapeutic sr-FS was lower for patients treated with RT compared to RPE in the “hormonal” domain, a finding corresponding to results of a study comparing external-beam RT with RPE [26]. However, our results must be interpreted with caution as they may reflect differential recruitment relative to treatment rather than differences between treatment groups. Furthermore, our study data do not allow for any conclusion on whether pretherapeutic sr-FS influences the choice of treatment. Many of our findings require further in-depth analysis—e.g., the large gradient relative to educational level in the sexual domain, which may in part be due to the patients’ desire to report better than actual sexual ability.

The robustness of the results was demonstrated in sensitivity analyses. In addition, the data allow unbiased calculation of the recruitment rate for each center [13], making adjustment possible for potential methodological artifacts that are typically not addressed. However, some limitations of the study need to be highlighted: recruitment varied widely across centers and relative to (1) the type of treatment administered (RT underrepresented), (2) insurance status (private insurance overrepresented), (3) school-leaving qualification (higher degrees overrepresented), and (4) age (young age overrepresented), potentially biasing the results [13]. Notably, since 16.8% of patients in the present study were classified as low-risk patients, the percentage of patients under AS (1.9%) appears to be particularly low. It may be argued that the selective recruitment relative to treatment in the present study is at least to some extent attributable to the restriction to patients receiving in-patient care [27]. In the present investigation, however, sensitivity analyses with adjustment for treatment choice, which may account for the selective recruitment, yielded results comparable to the main results. In this study, the quality of documentation in relation to missing values was good for the predictor variables—with the exception of the number of comorbidities since not all centers documented comorbidities. However, sensitivity analyses demonstrated the robustness of the results when only centers that documented comorbidities were taken into account. In addition, current knowledge does not allow any conclusions to be drawn regarding the clinical significance of effects on pretherapeutic FS scores reported in this study. However, the magnitude of effects appeared to be large for some associations (i.e., indicating a difference in the respective sr-FS score of > 5 points), suggesting that the corresponding predictors are clinically relevant [26]. On a positive note, information on education was collected and was shown to be of high importance for identifying patients with impaired pretherapeutic sr-FS, a finding that has been shown for clinical outcomes earlier [28]. In similar studies, unfortunately, data on socioeconomic status are often missing and are also not suggested by the ICHOM standard set.

Pretherapeutic sr-FS varies according to clinical patient characteristics and age but also with respect to socioeconomic status—as measured by educational status. This points out the benefit of collecting and considering socioeconomic information in addition to clinical and demographic patient characteristics for treatment decision-making and fair comparisons between health-care providers. Confirmation of the present findings in further well-powered studies would be desirable.

## Electronic supplementary material

Below is the link to the electronic supplementary material.

Supplementary file1 (DOCX 25 kb)

Supplementary file2 (DOCX 24 kb)

Supplementary file3 (DOCX 26 kb)

Supplementary file4 (DOCX 15 kb)

Supplementary file5 (DOCX 29 kb)

Supplementary file6 (DOCX 29 kb)

Supplementary file7 (DOCX 31 kb)

Supplementary file8 (DOCX 34 kb)

Supplementary file9 (DOCX 36 kb)

Supplementary file10 (DOCX 21 kb)
