# Supplementary material for: Determinants of self-reported functional status (EPIC-26) in prostate cancer patients prior to treatment
Source: World J Urol. 2020 Feb 10;39(1):27–36. doi: 10.1007/s00345-020-03097-z (PMC7858203; doi:10.1007/s00345-020-03097-z)
Supplement: Supplementary file 1 — Supplementary file1 (DOCX 25 kb) [file 345_2020_3097_MOESM1_ESM.docx]

Determinants of self-reported functional status (EPIC-26) in prostate cancer patients prior to treatment

**Rebecca Hein**^1,*^ · **Sebastian Dieng**^2^ · **Alisa Oesterle**^2^ · **Günter Feick^3^ · Günther Carl^4^ · Andreas Hinkel^5^ · Thomas Steiner^6^ · Björn Theodor Kaftan^7^ · Frank Kunath^8^ · Boris Hadaschik^9^ · Simba-Joshua Oostdam^10^ · Rein Jüri Palisaar^11^ · Mateusz Koralewski^12^ · Burkhard Beyer^13^ · Björn Haben^14^ ·** **Tsaur, Igor** ^15^ · **Simone Wesselmann**^16^ · **Christoph Kowalski**^15^

^1^ Institute of Medical Statistics and Computational Biology, Medical Faculty, University of Cologne, Germany; ^2^OnkoZert, Neu-Ulm, Germany; ^3^ Federal Association of German Prostate Cancer Patient Support Groups, Bonn, Germany; ^4^ Help for Prostate Cancer Patients (Förderverein Hilfe bei Prostatakrebs e.V., FHbP), Tornesch, Germany; ^5^ Franziskus Hospital, Bielefeld; ^6^ Helios Klinikum Erfurt; ^7^ Städtisches Klinikum Lüneburg; ^8^ Department of Urology and Pediatric Urology, University Hospital Erlangen, FAU Erlangen-Nürnberg; ^9^ Klinik und Poliklinik für Urologie,Kinderurologie und Uroonkologie, Universitätsklinikum Essen (AöR); ^10^ Vinzenz-Krankenhaus Hannover; ^11^ Urologische Klinik, Marien Hospital Herne; ^12^ Urologie, Krankenhaus der Barmherzigen Brüder Trier; ^13^ Martini-Klinik Prostate Cancer Center Hamburg; ^14^ St. Marien Hospital Ahaus; ^15^ Klinik und Poliklinik für Urologie und Kinderurologie, Universitätsmedizin der Johannes Gutenberg-Universität Mainz; ^16^ German Cancer Society, Berlin, Germany

Online Resource 1: Descriptive statistics for excluded patients (n=220) — pretherapeutic functional status and predictor variables

| **Pre-therapeutic functional status** |  | **Mean ± SD^a^ / Median (IQR^b^) / Range** |
| --- | --- | --- |
| Urinary incontinence (missing: 6.7% (N=1)) |  | 88.86±17.7 / 100 (85.5-100) / 8.25-100 |
| Urinary irritative/obstructive (missing: 6.7% (N=1)) |  | 82±18.89 / 87.5 (68.75-100) / 12.5-100 |
| Bowel (missing: 6.7% (N=1)) |  | 93.99±11.57 / 100 (91.67-100) / 25-100 |
| Sexual (missing: 6.7% (N=1)) |  | 48.62±30.66 / 50 (20.83-77.83) / 0-100 |
| Hormonal (missing: 6.7% (N=1)) |  | 87.65±15.78 / 95 (80-100) / 15-100 |
|  |  |  |
| **Patient characteristics** |  |  |
| ***Sociodemographic information*** |  | **Mean ± SD^a^ / Median (IQR^b^) / Range** |
| Age (missing: 0% (N=0)) |  | 68±8 / 69.5 (63-74) / 50-83 |
|  |  | **Percent (N)** |
| Citizenship (missing: 0% (N=0)) | German | 94.5 (208) |
|  | other | 2.3 (5) |
| Insurance (missing: 0% (N=0)) | statutory | 72.7 (160) |
|  | private | 19.1 (42) |
|  | other | 5 (11) |
| School-leaving qualification (missing: 0% (N=0)) | lower secondary school | 42.3 (93) |
|  | intermediate secondary school | 25 (55) |
|  | FHSR^c^ | 13.2 (29) |
|  | university entrance certificate | 13.2 (29) |
|  | other | 1.8 (4) |
|  | none | 1.8 (4) |
| ***Disease information*** |  | **Percent (N)** |
| Comorbidities (missing: 6.7% (N=1)) | 0 | 51.4 (113) |
|  | 1 | 15.9 (35) |
|  | >=2 | 6.4 (14) |
| Risk class^d^ (missing: 0% (N=0)) | high, localized | 30.5 (67) |
|  | intermediate, localized | 33.6 (74) |
|  | low, localized | 29.1 (64) |
|  | locally advanced | 3.6 (8) |
|  | advanced | 3.2 (7) |
| ***Treatment allocation*** |  |  |
| ADT^e^ (within three months after diagnosis) (missing: 0% (N=0)) | No | 53.6 (118) |
|  | Yes | 46.4 (102) |
| AS^f^ (within three months after diagnosis) (missing: 0% (N=0)) | No | 63.6 (140) |
|  | Yes | 36.4 (80) |
| WW^g^ (within three months after diagnosis) (missing: 0% (N=0)) | No | 93.6 (206) |
|  | Yes | 6.4 (14) |
| Treatment after questionnaire administration (missing: 0% (N=0)) | RPE^h^ | 47.3 (104) |
|  | RCP^i^ | 4.5 (10) |
|  | RT^j^ | 28.2 (62) |
|  | AS^f^ | 13.2 (29) |
|  | WW^g^ | 5 (11) |
|  | other | 1.8 (4) |
|  |  |  |
| **Center characteristics** |  | **Mean ± SD^a^ / Median (IQR ^b^) / Range** |
| Recruitment rate (missing: 0% (N=0)) |  | 43.53±19.56 / 38 (29-56) / 6-95 |
|  |  | **Percent (N)** |
| Ownership (missing: 0% (N=0)) | charitable | 8.6 (19) |
|  | for-profit | 43.2 (95) |
|  | public | 48.2 (106) |
| Urbanity (missing: 0% (N=0)) | ≤100T^k^ | 48.6 (107) |
|  | >100T^k^-1M^l^ | 48.2 (106) |
|  | >1M^l^ | 3.2 (7) |
| Teaching (missing: 0% (N=0)) | no | 2.7 (6) |
|  | yes, non-university | 82.3 (181) |
|  | yes, university | 15 (33) |

^a^ SD: standard deviation

^b^ IQR: interquartile range

^c^ FHSR: entrance certificate for a higher technical college/university of applied science

^d^ Risk class according to d’Amico (D'Amico et al., 1998)…

^e^ ADT: androgen deprivation therapy

^f^ AS: active surveillance

^g^ WW: watchful waiting

^h^ RPE: radical prostatectomy

^i^ RCP: radical cystectomy

^j^ RT: radiotherapy

^k^ T: thousand

^l^ M: million
