# Supplementary material for: Determinants of self-reported functional status (EPIC-26) in prostate cancer patients prior to treatment
Source: World J Urol. 2020 Feb 10;39(1):27–36. doi: 10.1007/s00345-020-03097-z (PMC7858203; doi:10.1007/s00345-020-03097-z)
Supplement: Supplementary file 4 — Supplementary file4 (DOCX 15 kb) [file 345_2020_3097_MOESM4_ESM.docx]

Determinants of self-reported functional status (EPIC-26) in prostate cancer patients prior to treatment

**Rebecca Hein**^1,*^ · **Sebastian Dieng**^2^ · **Alisa Oesterle**^2^ · **Günter Feick^3^ · Günther Carl^4^ · Andreas Hinkel^5^ · Thomas Steiner^6^ · Björn Theodor Kaftan^7^ · Frank Kunath^8^ · Boris Hadaschik^9^ · Simba-Joshua Oostdam^10^ · Rein Jüri Palisaar^11^ · Mateusz Koralewski^12^ · Burkhard Beyer^13^ · Björn Haben^14^ ·** **Tsaur, Igor** ^15^ · **Simone Wesselmann**^16^ · **Christoph Kowalski**^15^

^1^ Institute of Medical Statistics and Computational Biology, Medical Faculty, University of Cologne, Germany; ^2^OnkoZert, Neu-Ulm, Germany; ^3^ Federal Association of German Prostate Cancer Patient Support Groups, Bonn, Germany; ^4^ Help for Prostate Cancer Patients (Förderverein Hilfe bei Prostatakrebs e.V., FHbP), Tornesch, Germany; ^5^ Franziskus Hospital, Bielefeld; ^6^ Helios Klinikum Erfurt; ^7^ Städtisches Klinikum Lüneburg; ^8^ Department of Urology and Pediatric Urology, University Hospital Erlangen, FAU Erlangen-Nürnberg; ^9^ Klinik und Poliklinik für Urologie,Kinderurologie und Uroonkologie, Universitätsklinikum Essen (AöR); ^10^ Vinzenz-Krankenhaus Hannover; ^11^ Urologische Klinik, Marien Hospital Herne; ^12^ Urologie, Krankenhaus der Barmherzigen Brüder Trier; ^13^ Martini-Klinik Prostate Cancer Center Hamburg; ^14^ St. Marien Hospital Ahaus; ^15^ Klinik und Poliklinik für Urologie und Kinderurologie, Universitätsmedizin der Johannes Gutenberg-Universität Mainz; ^16^ German Cancer Society, Berlin, Germany

Online Resource 4: Intraclass correlation coefficients for linear multilevel models 1 to 3 and the null model

|  | Urinary incontinence | | Urinary irritative/obstructive | | Bowel | Sexual | Hormonal |
| --- | --- | --- | --- | --- | --- | --- | --- |
|  | ICC; p-value | | ICC; p-value | | ICC; p-value | ICC; p-value | ICC; p-value |
|  | |  | |  |  |  |  |
| Null model | 0.014;  0.059 | | 0.008;  0.154 | | <0.001;  0.581 | 0.024;  0.015 | 0.008;  0.134 |
| Model 1 | 0.007;  0.180 | | 0.007;  0.185 | | <0.001;  0.773 | 0.007;  0.163 | 0.007;  0.187 |
| Model 2 | 0.003;  0.336 | | 0.007;  0.198 | | <0.001;  0.964 | 0.002;  0.412 | 0.001;  0.531 |
| Model 3 | 0.005;  0.290 | | 0.009;  0.180 | | <0.001;  0.586 | 0.004;  0.357 | <0.001;  0.913 |
