# Supplementary material for: Determinants of self-reported functional status (EPIC-26) in prostate cancer patients prior to treatment
Source: World J Urol. 2020 Feb 10;39(1):27–36. doi: 10.1007/s00345-020-03097-z (PMC7858203; doi:10.1007/s00345-020-03097-z)
Supplement: Supplementary file 5 — Supplementary file5 (DOCX 29 kb) [file 345_2020_3097_MOESM5_ESM.docx]

Determinants of self-reported functional status (EPIC-26) in prostate cancer patients prior to treatment

**Rebecca Hein**^1,*^ · **Sebastian Dieng**^2^ · **Alisa Oesterle**^2^ · **Günter Feick^3^ · Günther Carl^4^ · Andreas Hinkel^5^ · Thomas Steiner^6^ · Björn Theodor Kaftan^7^ · Frank Kunath^8^ · Boris Hadaschik^9^ · Simba-Joshua Oostdam^10^ · Rein Jüri Palisaar^11^ · Mateusz Koralewski^12^ · Burkhard Beyer^13^ · Björn Haben^14^ ·** **Tsaur, Igor** ^15^ · **Simone Wesselmann**^16^ · **Christoph Kowalski**^15^

^1^ Institute of Medical Statistics and Computational Biology, Medical Faculty, University of Cologne, Germany; ^2^OnkoZert, Neu-Ulm, Germany; ^3^ Federal Association of German Prostate Cancer Patient Support Groups, Bonn, Germany; ^4^ Help for Prostate Cancer Patients (Förderverein Hilfe bei Prostatakrebs e.V., FHbP), Tornesch, Germany; ^5^ Franziskus Hospital, Bielefeld; ^6^ Helios Klinikum Erfurt; ^7^ Städtisches Klinikum Lüneburg; ^8^ Department of Urology and Pediatric Urology, University Hospital Erlangen, FAU Erlangen-Nürnberg; ^9^ Klinik und Poliklinik für Urologie,Kinderurologie und Uroonkologie, Universitätsklinikum Essen (AöR); ^10^ Vinzenz-Krankenhaus Hannover; ^11^ Urologische Klinik, Marien Hospital Herne; ^12^ Urologie, Krankenhaus der Barmherzigen Brüder Trier; ^13^ Martini-Klinik Prostate Cancer Center Hamburg; ^14^ St. Marien Hospital Ahaus; ^15^ Klinik und Poliklinik für Urologie und Kinderurologie, Universitätsmedizin der Johannes Gutenberg-Universität Mainz; ^16^ German Cancer Society, Berlin, Germany

Online Resource 5: Sensitivity analysis: Bootstrap results of multilevel analyses for pretherapeutic functional status (model 3)

|  | Urinary incontinence (2679 patients, 42 centers) | | Urinary irritative/obstructive (2612 patients, 42 centers) | | Bowel (2617 patients, 42 centers) | Sexual (2729 patients, 42 centers) | Hormonal (2678 patients, 42 centers) |
| --- | --- | --- | --- | --- | --- | --- | --- |
|  | Estimate (95% CI); p-value | | Estimate (95% CI); p-value | | Estimate (95% CI); p-value | Estimate (95% CI); p-value | Estimate (95% CI); p-value |
| (Intercept) | 104.34 (98.65-110.04) | | 95.65 (89.25-102.05) | | 95.47 (91.75-99.19) | 149.13 (138.8-159.47) | 75.45 (69.96-80.94) |
| **Patient characteristics** | |  | |  |  |  |  |
| *Sociodemographic information* | |  | |  |  |  |  |
| Age | **-0.19 (-0.27--0.11);**  **<0.001*** | | **-0.13 (-0.21--0.04);**  **0.004*** | | 0 (-0.05-0.06);  0.892 | **-1.39 (-1.53--1.24);**  **<0.001*** | **0.25 (0.17-0.32);**  **<0.001*** |
| Nationality |  | |  | |  |  |  |
| German | Reference | | Reference | | Reference | Reference | Reference |
| Other | -2.17 (-5.30-0.96);  0.175 | | -2.94 (-6.36-0.48);  0.092 | | 0.12 (-1.91-2.15);  0.908 | 0.45 (-5.39-6.29);  0.879 | 0.46 (-2.56-3.47);  0.767 |
| Insurance |  | |  | |  |  |  |
| Statutory | Reference | | Reference | | Reference | Reference | Reference |
| Private | 1.17 (-0.29-2.62);  0.116 | | 1.26 (-0.34-2.87);  0.122 | | 0.72 (-0.24-1.69);  0.143 | 2.41 (-0.22-5.05);  0.073 | 1.29 (-0.13-2.7);  0.076 |
| School-leaving qualification |  | |  | |  |  |  |
| Lower secondary school | Reference | | Reference | | Reference | Reference | Reference |
| Intermediate secondary school | 1.35 (-0.13-2.83);  0.073 | | 0.69 (-0.96-2.34);  0.411 | | 0.34 (-0.65-1.33);  0.504 | **3.45 (0.77-6.14);**  **0.012*** | **2.15 (0.69-3.61);**  **0.004*** |
| FHSR^a^ | 0.94 (-0.93-2.8);  0.325 | | -0.28 (-2.32-1.77);  0.791 | | -0.77 (-2.02-0.47);  0.224 | **6.42 (3.03-9.80);**  **<0.001*** | -0.11 (-1.97-1.75);  0.911 |
| University entrance certificate | 0.74 (-0.89-2.36);  0.373 | | 0.18 (-1.65-2.00);  0.850 | | 0.18 (-0.9-1.27);  0.744 | **8.34 (5.35-11.33);**  **<0.001*** | **2.00 (0.40-3.61);**  **0.015*** |
| Other | 0.66 (-3.94-5.26);  0.778 | | -2.48 (-7.56-2.60);  0.339 | | 0.94 (-2.03-3.91);  0.536 | -1.53 (-9.97-6.90);  0.722 | 1.54 (-2.97-6.06);  0.502 |
| None | -0.60 (-7.48-6.28);  0.864 | | **-10.46 (-17.88--3.04);**  **0.006*** | | **-4.73 (-9.08--0.38);**  **0.033** | -7.84 (-19.91-4.23);  0.203 | 0.79 (-5.78-7.36);  0.813 |
| *Disease information* | |  | |  |  |  |  |
| Comorbidities |  | |  | |  |  |  |
| 0 | Reference | | Reference | | Reference | Reference | Reference |
| 1 | 0.34 (-1.12-1.8);  0.649 | | 0.67 (-0.97-2.31);  0.425 | | -0.40 (-1.35-0.56); 0.416 | **-4.82 (-7.52--2.11);**  **<0.001*** | **-2.15 (-3.58--0.73);**  **0.003*** |
| ≥ 2 | 1.18 (-1.09-3.44);  0.309 | | -1.64 (-4.16-0.89);  0.204 | | **-1.71 (-3.20--0.21);**  **0.025*** | **-8.79 (-13.02--4.56);**  **<0.001*** | **-4.37 (-6.59--2.16);**  **<0.001*** |
| Risk class ^b^ |  | |  | |  |  |  |
| High, localized | -0.44 (-2.15-1.27);  0.616 | | **-2.11 (-4.00--0.22);**  **0.029*** | | **-1.34 (-2.49--0.19);**  **0.023*** | -0.92 (-4.07-2.24);  0.568 | **-1.99 (-3.71--0.27);**  **0.024*** |
| Intermediate, localized | 0.56 (-1.06-2.19);  0.497 | | -0.12 (-1.92-1.69);  0.898 | | 0.76 (-1.86-0.34);  0.177 | 1.84 (-1.14-4.82);  0.227 | -0.39 (-2.02-1.24);  0.638 |
| Low, localized | Reference | | Reference | | Reference | Reference | Reference |
| Locally advanced | 1.34 (-1.58-4.26);  0.368 | | **-3.46 (-6.70--0.22);**  **0.036*** | | 1.12 (-3.05-0.82);  0.258 | -1.02 (-6.24-4.20);  0.700 | -1.39 (-4.28-1.51);  0.347 |
| Advanced (N1) | -2.01 (-7.28-3.26);  0.455 | | **-13.38 (-19.33--7.42);**  **<0.001*** | | **-5.36 (-9.00--1.72);**  **0.004*** | -8.05 (-18.11-2.01);  0.117 | -0.94 (-6.11-4.22);  0.721 |
|  |  | |  | |  |  |  |
| **Center characteristics** | |  | |  |  |  |  |
| Recruitment rate | -1.14 (-5.14-2.86);  0.575 | | 0.05 (-4.76-4.86);  0.983 | | **2.61 (0.16-5.05);**  **0.036*** | -0.41 (-7.68-6.87);  0.912 | -2.11 (-5.68-1.46);  0.246 |
| Ownership |  | |  | |  |  |  |
| Public | Reference | | Reference | | Reference | Reference | Reference |
| Charitable | 0.20 (-4.03-4.43);  0.925 | | -1.33 (-6.24-3.58);  0.595 | | **-2.67 (-5.22--0.12);**  **0.040*** | -2.04 (-9.46-5.38);  0.590 | -2.74 (-6.48-0.99);  0.150 |
| For-profit | -0.16 (-2.12-1.80);  0.873 | | 0.11 (-2.19-2.41);  0.927 | | -1.00 (-2.16-0.15);  0.088 | -0.67 (-4.10-2.75);  0.700 | -0.59 (-2.28-1.10);  0.493 |
| Urban status |  | |  | |  |  |  |
| ≤ 100,000 | Reference | | Reference | | Reference | Reference | Reference |
| > 100,000–1,000,000 | 0.24 (-1.67-2.15);  0.805 | | -1.41 (-3.70-0.87);  0.226 | | **1.13 (0.01-2.26);**  **0.049*** | -0.32 (-3.68-3.04);  0.851 | 1.50 (-0.16-3.16);  0.077 |
| > 1,000,000 | -1.66 (-5.72-2.40);  0.423 | | -3.60 (-8.32-1.13);  0.136 | | 0.79 (-1.67-3.25);  0.528 | 0.00 (-7.36-7.36);  0.999 | 2.54 (-1.16-6.23);  0.179 |
| Teaching |  | |  | |  |  |  |
| No | 0.41 (-2.90-3.73);  0.806 | | **-4.08 (-7.95--0.20);**  **0.039*** | | 1.28 (-0.75-3.31);  0.218 | 2.99 (-8.84-2.86);  0.316 | -0.91 (-3.89-2.08);  0.551 |
| Yes, non-university | Reference | | Reference | | Reference | Reference | Reference |
| Yes, university | -1.18 (-3.79-1.44);  0.378 | | 0.38 (-2.73-3.48);  0.813 | | **-1.55 (-3.03--0.07);**  **0.040*** | -1.21 (-5.79-3.37);  0.605 | -0.94 (-3.14-1.26);  0.404 |

Note: (1) Multilevel model 3 accounts for the clustering of patients (level 1) in PCCs (level 2) with a random intercept. Fixed effects for sociodemographic predictors, information on disease and treatment allocation and PCC characteristics are included. (2) Results regarding the missing categories for categorical variables are not presented in this table. (3) Results are based on 10,000 bootstrap samples.

^a^ FHSR: entrance certificate for a higher technical college/university of applied science

^b^ Risk class according to d’Amico (D'Amico et al., 1998)

*P < 0.05
