# Supplementary material for: Determinants of self-reported functional status (EPIC-26) in prostate cancer patients prior to treatment
Source: World J Urol. 2020 Feb 10;39(1):27–36. doi: 10.1007/s00345-020-03097-z (PMC7858203; doi:10.1007/s00345-020-03097-z)
Supplement: Supplementary file 9 — Supplementary file9 (DOCX 36 kb) [file 345_2020_3097_MOESM9_ESM.docx]

Determinants of self-reported functional status (EPIC-26) in prostate cancer patients prior to treatment

**Rebecca Hein**^1,*^ · **Sebastian Dieng**^2^ · **Alisa Oesterle**^2^ · **Günter Feick^3^ · Günther Carl^4^ · Andreas Hinkel^5^ · Thomas Steiner^6^ · Björn Theodor Kaftan^7^ · Frank Kunath^8^ · Boris Hadaschik^9^ · Simba-Joshua Oostdam^10^ · Rein Jüri Palisaar^11^ · Mateusz Koralewski^12^ · Burkhard Beyer^13^ · Björn Haben^14^ ·** **Tsaur, Igor** ^15^ · **Simone Wesselmann**^16^ · **Christoph Kowalski**^15^

^1^ Institute of Medical Statistics and Computational Biology, Medical Faculty, University of Cologne, Germany; ^2^OnkoZert, Neu-Ulm, Germany; ^3^ Federal Association of German Prostate Cancer Patient Support Groups, Bonn, Germany; ^4^ Help for Prostate Cancer Patients (Förderverein Hilfe bei Prostatakrebs e.V., FHbP), Tornesch, Germany; ^5^ Franziskus Hospital, Bielefeld; ^6^ Helios Klinikum Erfurt; ^7^ Städtisches Klinikum Lüneburg; ^8^ Department of Urology and Pediatric Urology, University Hospital Erlangen, FAU Erlangen-Nürnberg; ^9^ Klinik und Poliklinik für Urologie,Kinderurologie und Uroonkologie, Universitätsklinikum Essen (AöR); ^10^ Vinzenz-Krankenhaus Hannover; ^11^ Urologische Klinik, Marien Hospital Herne; ^12^ Urologie, Krankenhaus der Barmherzigen Brüder Trier; ^13^ Martini-Klinik Prostate Cancer Center Hamburg; ^14^ St. Marien Hospital Ahaus; ^15^ Klinik und Poliklinik für Urologie und Kinderurologie, Universitätsmedizin der Johannes Gutenberg-Universität Mainz; ^16^ German Cancer Society, Berlin, Germany

Online Resource 9: Sensitivity analysis: Results of linear multilevel analyses for pretherapeutic functional status (model 3) with adjustment for treatment after questionnaire administration

|  | Urinary incontinence (2865 patients, 42 centers) | | Urinary irritative/obstructive (2789 patients, 42 centers) | | Bowel (2802 patients, 42 centers) | Sexual (2923 patients, 42 centers) | Hormonal (2866 patients, 42 centers) |
| --- | --- | --- | --- | --- | --- | --- | --- |
|  | Estimate (95% CI); p-value | | Estimate (95% CI); p-value | | Estimate (95% CI); p-value | Estimate (95% CI); p-value | Estimate (95% CI); p-value |
| (Intercept) | 103.74 (98.05-109.43) | | 95.52 (89.19-101.85) | | 95.43 (91.73-99.14) | 147.92 (137.54-158.3) | 74.57 (69.05-80.09) |
| **Patient characteristics** | |  | |  |  |  |  |
| *Sociodemographic information* | |  | |  |  |  |  |
| Age | **-0.18 (-0.26--0.10);**  **<0.001*** | | **-0.13 (-0.21--0.04);**  **0.005*** | | 0.00 (-0.05-0.05);  0.922 | **-1.36 (-1.51--1.22);**  **<0.001*** | **0.27 (0.19-0.34);**  **<0.001*** |
| Nationality |  | |  | |  |  |  |
| German | Reference | | Reference | | Reference | Reference | Reference |
| Other | -2.18 (-5.29-0.93);  0.170 | | -2.87 (-6.3-0.56);  0.101 | | 0.13 (-1.92-2.17);  0.904 | 0.30 (-5.47-6.07);  0.918 | 0.34 (-2.69-3.37);  0.826 |
| Insurance |  | |  | |  |  |  |
| Statutory | Reference | | Reference | | Reference | Reference | Reference |
| Private | 1.12 (-0.32-2.56);  0.128 | | 1.25 (-0.35-2.84);  0.125 | | 0.72 (-0.24-1.67);  0.142 | 2.33 (-0.32-4.97);  0.085 | 1.22 (-0.2-2.63);  0.093 |
| School-leaving qualification |  | |  | |  |  |  |
| Lower secondary school | Reference | | Reference | | Reference | Reference | Reference |
| Intermediate secondary school | 1.4 (-0.08-2.89);  0.064 | | 0.76 (-0.88-2.41);  0.362 | | 0.31 (-0.68-1.3);  0.543 | **3.44 (0.73-6.14);**  **0.013*** | **2.21 (0.74-3.67);**  **0.003*** |
| FHSR  ^a^ | 0.93 (-0.93-2.78);  0.327 | | -0.21 (-2.25-1.84);  0.844 | | -0.8 (-2.04-0.45);  0.211 | **6.32 (2.92-9.73);**  **<0.001*** | -0.1 (-1.95-1.75);  0.912 |
| University entrance certificate | 0.82 (-0.81-2.45);  0.326 | | 0.29 (-1.52-2.09);  0.754 | | 0.14 (-0.94-1.23);  0.795 | **8.29 (5.29-11.29);**  **<0.001*** | **2.01 (0.4-3.61);**  **0.014*** |
| Other | 0.65 (-3.92-5.21);  0.781 | | -2.52 (-7.57-2.52);  0.327 | | 0.84 (-2.11-3.8);  0.576 | -1.42 (-9.77-6.93);  0.739 | 1.77 (-2.71-6.24);  0.439 |
| None | -0.47 (-7.3-6.35);  0.892 | | **-10.3 (-17.75--2.85);**  **0.007*** | | **-4.68 (-8.99--0.38);**  **0.033*** | -7.93 (-20-4.13);  0.198 | 0.66 (-5.96-7.28);  0.845 |
| *Disease information* | |  | |  |  |  |  |
| Comorbidities |  | |  | |  |  |  |
| 0 | Reference | | Reference | | Reference | Reference | Reference |
| 1 | 0.41 (-1.04-1.86);  0.579 | | 0.79 (-0.84-2.42);  0.342 | | -0.36 (-1.31-0.59);  0.454 | **-4.87 (-7.54--2.21);**  **<0.001*** | **-2.3 (-3.72--0.89);**  **0.001*** |
| ≥ 2 | 1.45 (-0.80-3.70);  0.208 | | -1.32 (-3.83-1.19);  0.303 | | **-1.62 (-3.1--0.14);**  **0.032*** | **-8.84 (-13--4.67);**  **<0.001*** | **-4.61 (-6.82--2.39);**  **<0.001*** |
| Risk class ^a^ |  | |  | |  |  |  |
| High, localized | -0.83 (-2.58-0.91);  0.350 | | **-2.35 (-4.28--0.42);**  **0.017*** | | **-1.29 (-2.46--0.12);**  **0.031*** | -1.21 (-4.42-1.99);  0.457 | **-2.26 (-3.98--0.53);**  **0.010*** |
| Intermediate, localized | 0.22 (-1.42-1.86);  0.792 | | -0.32 (-2.15-1.5);  0.730 | | -0.73 (-1.83-0.37);  0.192 | 1.61 (-1.41-4.63);  0.296 | -0.59 (-2.22-1.04);  0.475 |
| Low, localized | Reference | | Reference | | Reference | Reference | Reference |
| Locally advanced | 0.93 (-2.01-3.88);  0.534 | | **-3.67 (-6.92--0.42);**  **0.027*** | | -1.07 (-3.02-0.88);  0.281 | -1.34 (-6.67-4);  0.623 | -1.64 (-4.58-1.29);  0.272 |
| Advanced (N1) | -2.61 (-7.98-2.77);  0.342 | | **-13.83 (-19.81--7.85);**  **<0.001*** | | **-5.32 (-8.94--1.71);**  **0.004*** | -8.35 (-18.33-1.63);  0.101 | -1.10 (-6.28-4.07);  0.676 |
|  | | | | | | | |
| *Treatment initiated after questionnaire administration* | | | | | | | |
| RPE ^c^ | Reference | | Reference | | Reference | Reference | Reference |
| RT ^d^ | 1.15 (-1.36-3.66);  0.369 | | 2.78 (-0.04-5.6);  0.053 | | 0.57 (-1.14-2.28);  0.514 | -2.86 (-7.61-1.9);  0.239 | **-3.18 (-5.69--0.67);**  **0.013*** |
| AS ^e^ | **-6.54 (-11.96--1.12);**  **0.018*** | | **-7.09 (-12.92--1.25);**  **0.017*** | | 2.27 (-1.42-5.96);  0.227 | 1.71 (-8.32-11.73);  0.739 | -1.41 (-6.77-3.95);  0.606 |
| WW ^f^ | **-16.4 (-27.29--5.5);**  **0.003*** | | -9.75 (-22.62-3.12);  0.138 | | **-8.5 (-15.7--1.3);**  **0.021*** | -17.72 (-36.67-1.24);  0.067 | -1.1 (-11.89-9.69);  0.841 |
|  |  | |  | |  |  |  |
| **Center characteristics** | |  | |  |  |  |  |
| Recruitment rate | -0.55 (-4.52-3.41);  0.784 | | 0.31 (-4.34-4.96);  0.896 | | **2.76 (0.34-5.18);**  **0.025*** | 0.51 (-6.49-7.51);  0.886 | -1.79 (-5.34-1.76);  0.32386841 |
| Ownership |  | |  | |  |  |  |
| Public | Reference | | Reference | | Reference | Reference | Reference |
| Charitable | 0.14 (-4-4.28);  0.948 | | -1.53 (-6.31-3.26);  0.532 | | **-2.71 (-5.22--0.19);**  **0.035*** | -1.87 (-9.12-5.37);  0.612 | -2.63 (-6.34-1.08);  0.165 |
| For-profit | 0.04 (-1.85-1.93);  0.968 | | 0.39 (-1.84-2.62);  0.730 | | -1.04 (-2.18-0.1);  0.074 | -0.65 (-3.97-2.66);  0.699 | -0.64 (-2.32-1.04);  0.458 |
| Urban status |  | |  | |  |  |  |
| ≤ 100,000 | Reference | | Reference | | Reference | Reference | Reference |
| > 100,000–1,000,000 | -0.18 (-2.06-1.69);  0.848 | | -1.82 (-4.04-0.4);  0.109 | | 1.10 (-0.02-2.23);  0.055 | -0.41 (-3.69-2.86);  0.804 | 1.56 (-0.1-3.22);  0.065 |
| > 1,000,000 | -1.99 (-5.96-1.99);  0.328 | | -3.86 (-8.45-0.73);  0.099 | | 0.84 (-1.59-3.28);  0.498 | -0.18 (-7.37-7.01);  0.960 | 2.45 (-1.25-6.16);  0.195 |
| Teaching |  | |  | |  |  |  |
| No | 0.98 (-2.29-4.25);  0.556 | | -3.39 (-7.2-0.42);  0.081 | | 1.28 (-0.76-3.33);  0.219 | -3.11 (-8.9-2.68);  0.292 | -0.90 (-3.89-2.09);  0.555 |
| Yes, non-university | Reference | | Reference | | Reference | Reference | Reference |
| Yes, university | -0.85 (-3.38-1.68);  0.512 | | 0.79 (-2.21-3.79);  0.608 | | **-1.57 (-3.05--0.10);**  **0.037*** | -1.43 (-5.84-2.97);  0.524 | -1.07 (-3.25-1.12);  0.339 |

Note: (1) Multilevel model 3 accounts for the clustering of patients (level 1) in PCCs (level 2) with a random intercept. Fixed effects for sociodemographic predictors, information on disease and treatment allocation and PCC characteristics are included. (2) Patient numbers vary between analyses of different scores due to the different number of missing values for the respective scores. (3) Results regarding the missing categories for categorical variables are not presented in this table. (4) These sensitivity analyses for model 3 were conducted after exclusion of centers that did not document comorbidities. Note that after exclusion of these centers, there were no more centers with charitable ownership included.

^a^ FHSR: entrance certificate for a higher technical college/university of applied science

^b^ Risk class according to d’Amico (D'Amico et al., 1998)

^c^ RPE: radical prostatectomy

^d^ RT: radiotherapy

^e^ AS: active surveillance

^f^ WW: watchful waiting

*P < 0.05
