# Supplementary material for: Determinants of self-reported functional status (EPIC-26) in prostate cancer patients prior to treatment
Source: World J Urol. 2020 Feb 10;39(1):27–36. doi: 10.1007/s00345-020-03097-z (PMC7858203; doi:10.1007/s00345-020-03097-z)
Supplement: Supplementary file 10 — Supplementary file10 (DOCX 21 kb) [file 345_2020_3097_MOESM10_ESM.docx]

Determinants of self-reported functional status (EPIC-26) in prostate cancer patients prior to treatment

**Rebecca Hein**^1,*^ · **Sebastian Dieng**^2^ · **Alisa Oesterle**^2^ · **Günter Feick^3^ · Günther Carl^4^ · Andreas Hinkel^5^ · Thomas Steiner^6^ · Björn Theodor Kaftan^7^ · Frank Kunath^8^ · Boris Hadaschik^9^ · Simba-Joshua Oostdam^10^ · Rein Jüri Palisaar^11^ · Mateusz Koralewski^12^ · Burkhard Beyer^13^ · Björn Haben^14^ ·** **Tsaur, Igor** ^15^ · **Simone Wesselmann**^16^ · **Christoph Kowalski**^15^

^1^ Institute of Medical Statistics and Computational Biology, Medical Faculty, University of Cologne, Germany; ^2^OnkoZert, Neu-Ulm, Germany; ^3^ Federal Association of German Prostate Cancer Patient Support Groups, Bonn, Germany; ^4^ Help for Prostate Cancer Patients (Förderverein Hilfe bei Prostatakrebs e.V., FHbP), Tornesch, Germany; ^5^ Franziskus Hospital, Bielefeld; ^6^ Helios Klinikum Erfurt; ^7^ Städtisches Klinikum Lüneburg; ^8^ Department of Urology and Pediatric Urology, University Hospital Erlangen, FAU Erlangen-Nürnberg; ^9^ Klinik und Poliklinik für Urologie,Kinderurologie und Uroonkologie, Universitätsklinikum Essen (AöR); ^10^ Vinzenz-Krankenhaus Hannover; ^11^ Urologische Klinik, Marien Hospital Herne; ^12^ Urologie, Krankenhaus der Barmherzigen Brüder Trier; ^13^ Martini-Klinik Prostate Cancer Center Hamburg; ^14^ St. Marien Hospital Ahaus; ^15^ Klinik und Poliklinik für Urologie und Kinderurologie, Universitätsmedizin der Johannes Gutenberg-Universität Mainz; ^16^ German Cancer Society, Berlin, Germany

Online Resource 10: Staging according to the German Guideline Prostate Cancer (version April 2018)

In this study we use the staging proposed by the German Guideline for Prostate Cancer [1]: **Localized prostate cancer**: T1-2, N0 M0

- - **Localized prostate cancer with low risk**: PSA ≤ 10ng/ml and Gleason-Score 6 and cT1c or cT2a
  - **Localized prostate cancer with intermediate risk**: PSA >10ng/ml – 30ng/ml or Gleason 7 or cT 2b
  - **Localized prostate cancer with high risk**: PSA > 20ng/ml or Gleason ≥ 8 or cT2c
- **Locally advanced prostate cancer:** T3-4 N0 M0
- **Advanced prostate cancer:** any T N1 and M0
- **Metastasized prostate cancer**: any T any N and M1

Furthermore, localized prostate cancer with cT1a or cT1b is classified as localized prostate cancer with low risk.

1. Leitlinienprogramm Onkologie (Deutsche Krebsgesellschaft, D.K., AWMF), *[Interdisziplinäre Leitlinie der Qualität S3 zur Früherkennung, Diagnose und Therapie der verschiedenen Stadien des Prostatakarzinoms, Langversion 5.0], p. 60*. 2018.
